# Finite element/percolation theory modelling of the micromechanical behavior of clayey soils

**DOI:** 10.1186/s40064-015-0887-9

**Published:** 2015-03-25

**Authors:** M Luz Pérez-Rea, Jaime Horta-Rangel, Teresa López-Lara, Juan B Hernández-Zaragoza, Sergio M Alcocer, Victor M Castaño

**Affiliations:** Universidad Autónoma de Querétaro, Facultad de Ingeniería, Cerro de las Campanas S/N, Col. Niños Héroes, 76010 Querétaro, Querétaro México; Centro de Física Aplicada y Tecnología Avanzada, Universidad Nacional Autónoma de México, Boulevard Juriquilla 3001, 76230 Querétaro, Querétaro México; Instituto de Ingeniería, Universidad Nacional Autónoma de México, Cd. Universitaria, México, D.F. 04510 México

## Abstract

A hybrid model for soils, which combines percolation theory and finite element method is presented. The internal soil structure is modelled via the finite element method, and percolation networks are used for analyzing its mechanical behaviour. Through a microscopic characterization of elastic properties of soil grains, the model is generated. The effective percolation threshold obtained is lower than that of the network geometric percolation. The effective mechanical properties predicted are successfully compared to published experimental results.

## 1. Introduction

In civil engineering practice, building foundations design involves the best-possible estimation of the effective mechanical properties, such as soil modulus of elasticity and Poisson ratio, for predicting the final building settlements. In recent studies, there has been an attempt to relate hydraulic conductivity and other properties of fine soils to changes in soil structure at three different levels, namely, micro-, mini-, and macro-structure. The first considers the small pores among single particles; the second, includes spacing among pores and clots; and the third incorporates macropores, such as cracks, fissures, root voids, and visible pores (Mitchel et al. 1973; Collis and McGown 1974; Reddi and Thangavadivelu 1996). Thus, at the microstructure level, flow occurs through macropores and medium-size features may be unimportant. Nevertheless, meso- and micro-pores flow involves intermolecular forces rising from a particular geometry or from electronic properties of the medium or fluid (Knutson andSelker 1994; Hsu and Masten 2001; McNamara and Luthy 2005; Guo and Chorover 2006). Flow, as other conductive properties of porous materials, becomes dependent of the nature of the components, geometric layout and internal conductivity. In this context, physisorption is a general phenomenon that occurs whenever an adsorptive liquid or gas is brought into contact with the surface of a solid (the adsorbent), involving forces of the same kind as those responsible for the condensation of vapors (Sing et al. 1985). In addition to the attractive dispersion forces and the short-range repulsive forces, specific molecular interactions (e.g. polarization, field-dipole, field gradient-quadrupole) usually occur, influencing the effective mechanical behavior of the materials. Because of the above, soil structure and its composition, at a microscopic level, are important properties to consider for developing models of mechanical behavior to attempt predicting real behavior. In this context, in the last thirty years a family of powerful theoretical methods have been developed; these models have allowed the interpretation of experimental results and the prediction of many properties of disordered micro- and macroscopic systems with regular and non-regular structures (Sahimi 1994). Among others, percolation theory has played an important role for understanding these systems and their properties. Specifically, problems related to porous media have received particular attention. For instance, bi-phase flow problems have been understood and at least, under some conditions, can be interpreted as a percolation phenomenon. Statistical Physics of disordered systems has as an aim to provide methods such that from macroscopic properties of such systems to derive laws at microscopic level. Alternatively, to deduce microscopic properties from macroscopic information observed via experimental techniques. The fast development of such theories is due to the fact that it seems that there is a relation between microscopic elements and its effects on the macroscopic properties. Most of the works reported in the literature deal with problems of hydraulic and electric conductivity. However, there are conductive properties that could be modeled as the elastic properties. In addition, literature reports on materials with repeated octahedral cells have shown that the finite element method (FEM) can be applied to predict effective elastic properties for porous materials such as metallic foams (Deshpande et al. 2001). Micromechanical models using properties such as grain size distribution, individual behavior or random structures of different kinds of composites have been realized using FEM with good results ((Berbenni et al. 2007; Prahl et al. 2007); Giannopoulos et al. In press). Perhaps, continuum mechanics cannot be applied to the modeling of materials behavior to macroscopic levels, but the single mechanical behavior of materials components could be modeled with this microscopic viewpoint. Thus, a combination of the percolation theory and the finite element method could lead to a useful prediction of the effective mechanical properties of fine soils.

## 2. Elastic properties of percolating systems

The first approximation to the elastic behaviour of random percolating nets was based on the problem analogous to the electrical conductivity in gels (De Gennes 1976). This, in turn, is based on Born’s model (Born and Huang 1954) for the microscopic elasticity of a cell. Considering a simple cubic lattice with central forces only, the shear modulus is zero and the bulk modulus goes to zero when an infinitesimally small fraction of bonds are missing (Feng and Sen 1984). In this case, the rigidity threshold is at *p = 1*. Feng and Seng studied numerically a 2D triangular lattice subjected to a hydrostatic pressure, finding that the elasticity exponent value is numerically greater than the conductivity exponent. However, it has been shown that the problem of percolation associated with this type of lattice elasticity is different from the one associated to regular site or bond percolation (Giannopoulos et al. In press) (Kantor and Webman 1984). Kantor and Webman (De Gennes 1976) proposed a model for the elasticity of a percolating network. In that model, the stiffness threshold was identical to the geometric one. The structure of the infinite percolating cluster has been analyzed via an elastic kinematic model. From that, the critical exponent **τ** was obtained. Results showed that the elastic behaviour of a percolating net belongs to a class universally different fromconductivity and that the critical exponent τ is much greater than the conductivity exponent *t*. The lattice model provides a correct description of the elastic behaviour of macroscopically inhomogeneous composed materials, built with regions locally stiff (solids) and regions locally very soft (voids). Close to *p*_*c*_, the material macroscopic stiffness is determined by the elasticity of long and tortuous channels of rigid material, which are in the main skeleton of the percolating cluster.

## 3. Finite element model (FEM) applied to networks representing soil

The material to be modelled herein is soil, but the methodology may be applied to any other porous media. Traditionally, soil behaviour has been modelled via elasticity theory with acceptable results. Soil basic properties may be determined using characterization techniques such as electron microscopy, X-ray diffraction and thermal analysis. When these are correctly interpreted, model developed closely represents soil real conditions. Once soil porosity has been modelled via percolation theory, the FEM can be applied for the analysis of the mechanical behaviour. Micromechanics analysis facilitates the comparison between results obtained via FEM and the real ones. For the bond percolation systems, and for the sake of simplicity, two bi-dimensional regular networks were used, namely, squared and hexagonal or panaloid. The squared network was used because is frequently encountered in different models, while the hexagonal network was used because it is more similar to soil microstructure, according to observations made via electron microscopy (Pérez-Rea 2005). Each bond represents single clay particles in three dimensions. Typically, two of them are small and the other is larger than 1 and 10 microns, respectively. Microscopic dimensions were determined through scan electron microscopy. The assumption of a rectangular section is based on the clay particles having a plate shape; pores are assumed to have flat walls. This was inferred from the adsorption isotherm form in the characterization of porosimetry with liquid nitrogen (Pérez-Rea et al. 2003) . Both networks provide a right description of the elastic behaviour of a macroscopic inhomogeneous composed material. This was assumed to be formed of locally rigid and soft regions (these soft regions represent soil voids). Each clay particle (montmorillonite, in this case) has its own properties, reported elsewhere (Lee 2002) , such as the elastic modulus *E* = 150 GPa and Poisson ratio ν = 0.35. Considering that clay grains are practically indestructible, due to the ionic bonds with which clay layers are linked (Keedwell 1984) , each grain can be assumed to behave as an elastic beam. This cannot necessarily reflect in the soil macroscopic behaviour. The main assumed hypothesis is that the transmission of forces inside the soil mass happens, at microscopic level, through the contact points of grains. These may be considered as rigid joints in soils with low humidity content based on the abovementioned hypothesis that has been proved macroscopically in granular soils (Skempton 1961) and, under the assumption that all pores are interconnected. There is a two-phase composite, i.e. a solid phase made of clay grains in different structures, and a void phase being the air inside pores. This assumption is justified since forces being transmitted from a building to a non-saturated soil are taken by the solid soil part and deformation effects associated to the difficulty of water elimination from pores are minimum compared to initial deformations. In this system, the expected stiffness threshold must be identical to the geometric percolation threshold of the rigid phase. Close to *p*_*cr*_, the material macroscopic stiffness will be determined by long and contorted channels of rigid material that belong to the percolating aggregate. The total external force will be taken by the aggregate skeleton. The probability of a bond being present, *p*, represents the soil solid part, and *1-p* represents the porosity. This can also be called the “dead” part of the structure, since it does not contributes to the system deformation strength. Networks can be modelled to analyze their behaviour stress-deformation by FEM. The commercial package for this analysis used was ANSYS^MR^ version 7.1. Elastic beam elements were used for the modelling, since it has capacity for tension, compression and bending. Each element has three degrees-of-freedom at each node, namely, two nodal displacements along the *x*- and *y*-axis and a rotation around *z*. For the loading condition, temperature was assumed to be constant. The element is defined by two nodes, the cross sectional area, *A*, the moment of inertia *I*, the section height, the modulus of elasticity *E,* and the Poisson ratioν. The length of the element, *L*, is defined by the distance between nodes I and J. The stiffness matrix *K*_*l*_ is given by (Przemieniecki 1968)

1 $$ \left[{K}_l\right]=\left[\begin{array}{cccccc}\hfill \frac{AE}{L}\hfill & \hfill 0\hfill & \hfill 0\hfill & \hfill -\frac{AE}{L}\hfill & \hfill 0\hfill & \hfill 0\hfill \\ {}\hfill 0\hfill & \hfill \frac{12EI}{L^3\left(1+\varphi \right)}\hfill & \hfill \frac{6EI}{L^2\left(1+\varphi \right)}\hfill & \hfill 0\hfill & \hfill -\frac{12EI}{L^3\left(1+\varphi \right)}\hfill & \hfill -\frac{6EI}{L^2\left(1+\varphi \right)}\hfill \\ {}\hfill 0\hfill & \hfill \frac{6EI}{L^2\left(1+\varphi \right)}\hfill & \hfill \frac{EI\left(4+\varphi \right)}{L\left(1+\varphi \right)}\hfill & \hfill 0\hfill & \hfill -\frac{6EI}{L^2\left(1+\varphi \right)}\hfill & \hfill \frac{EI\left(2-\varphi \right)}{L\left(1+\varphi \right)}\hfill \\ {}\hfill -\frac{AE}{L}\hfill & \hfill 0\hfill & \hfill 0\hfill & \hfill \frac{AE}{L}\hfill & \hfill 0\hfill & \hfill 0\hfill \\ {}\hfill 0\hfill & \hfill -\frac{12EI}{L^3\left(1+\varphi \right)}\hfill & \hfill -\frac{6EI}{L^2\left(1+\varphi \right)}\hfill & \hfill 0\hfill & \hfill \frac{12EI}{L^3\left(1+\varphi \right)}\hfill & \hfill -\frac{6EI}{L^2\left(1+\varphi \right)}\hfill \\ {}\hfill 0\hfill & \hfill \frac{6EI}{L^2\left(1+\varphi \right)}\hfill & \hfill \frac{EI\left(2-\varphi \right)}{L\left(1+\varphi \right)}\hfill & \hfill 0\hfill & \hfill -\frac{6EI}{L^2\left(1+\varphi \right)}\hfill & \hfill \frac{EI\left(4+\varphi \right)}{L\left(1+\varphi \right)}\hfill \end{array}\right] $$

The shape factor ϕ is defined by

$$ \varphi =\frac{12EI}{G{A}^s{L}^2} $$

where *G* is the shear?? modulus of rigidity and *A*^*s*^ = *A*/*F*^*s*^, the so-called shear area, and *F*^*s*^ is the shear deflection constant.

Although the problem was thought of as a 2D truss for the loading analysis, the deflections analysis in each element takes into account the structural sections of beams. This is the way for realizing analysis to structural frames of buildings. The cross section was considered squared of 1 micron per side. The beam elements have a length of 10 microns. Each line between nodes is a beam element and it is equivalent to a net loop. For the modelling of nets with different porosities, APDL, from ANSYS^MR^, was used.

### Simulation procedure

Full bonds networks were modeled with only one material. This material, called henceforth “M1”, had the elastic properties of clay grains and its probability of being occupied is *p = 1*. The grain size distribution was omitted in these models; however, characterizations at microscopic levels were made to determine the mean grain size (TMG) for modeling regular nets. A second material, called henceforth “M2”, with identical mechanical properties of M1 was introduced. This material represents the soil voids, i.e. the pores.

Starting with an occupied probability *p*, net bonds were randomly selected and changed from M1 to M2. Thus, a network composed of bonds of two different materials, but with identical mechanical properties is modeled. M1 occupies a fraction *p* of bonds of the network whereas M2 occupies a fraction 1-*p* of the same.

To minimize the influence on the mechanical behavior of the network, the M2 stiffness represented by the elastic modulus was reduced in a 1×10^−8^ scale. The bonds of this material become extremely soft within a rigid structure whose resisting skeleton are all M1 bonds. The stiffness of M2 must be selected close to zero to model pores network, although structurally those bonds exist. The mechanical analysis is done via FEM. Nets were subjected to axial vertical compression, varying from zero up to the value needed to fail the structure due to excessive deformation. All the nodes had free displacements, such as it happens in lab tests, except the lower nodes that were constrained in the three directions.

Results were analyzed to find the mean value of vertical and horizontal strains, as well as the Poisson ratio. All load-strain processes were analyzed in order to obtain the modulus of elasticity of each of the net models, thus obtaining a mean modulus of elasticity for each value of porosity at each net.

## 4. Results and discussion

**Figure 1:**
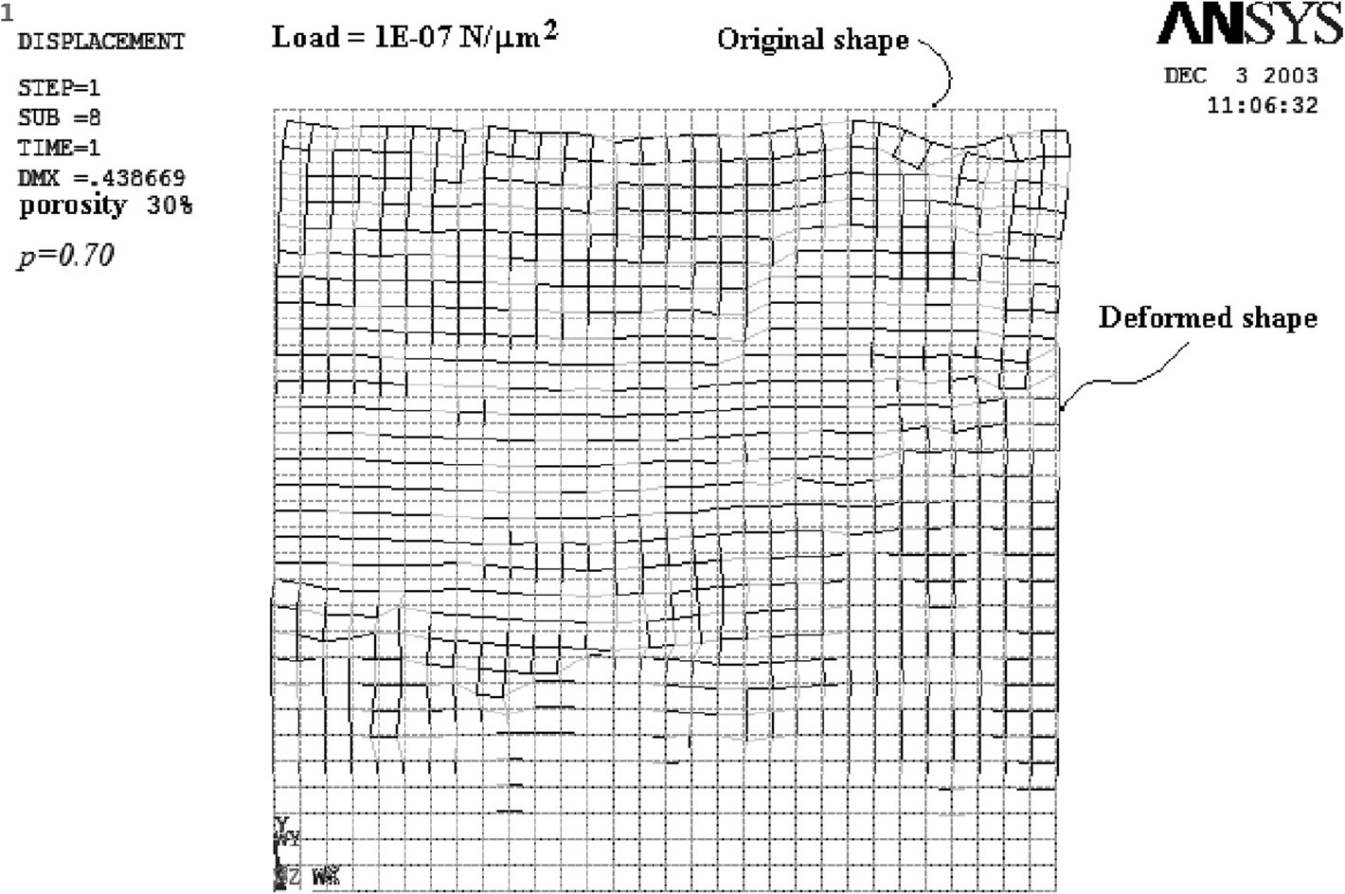
**FEM results of the squared net for a percolation factor** ***p =*** **0.70.**

**Figure 2:**
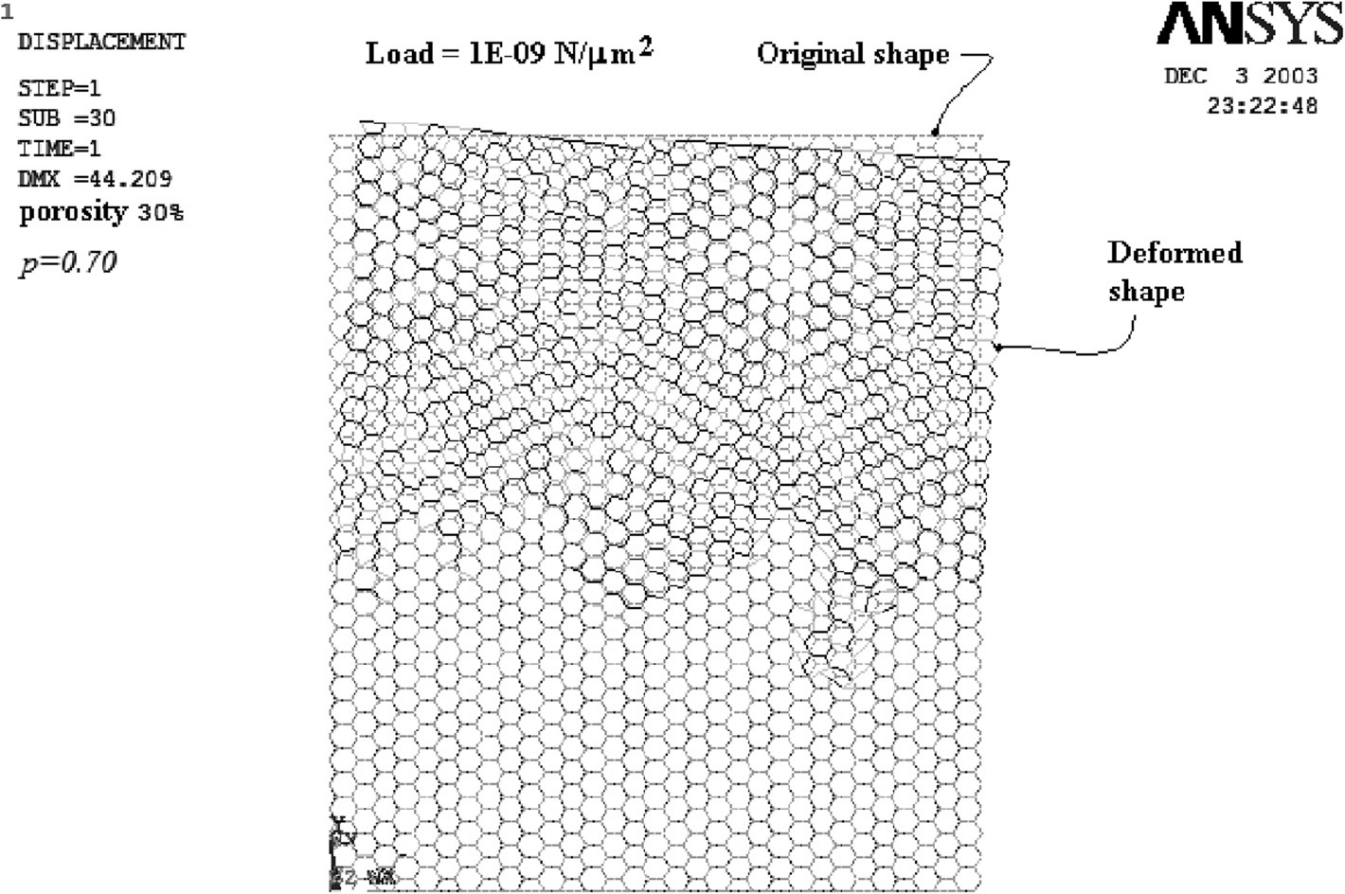
**FEM results for the hexagonal net for a percolation factor** ***p =*** **0.70.**

Deformed rectangular and hexagonal nets after FEM analysis with vertical load application are shown in Figures 1 and 2, respectively. The original configuration network is drawn with gray dashed lines, whereas the deformed net has been superposed in black color. For each network, several runs were carried out.

Based on the above, the mechanical behavior was modeled. By using the elasticity critical exponent τ = 2.0549 (De Gennes 1976) for 2D and considering that the macroscopic stiffness, E*, of the main aggregate is given by

2 $$ E\ast =E{\left(p-{p}_{cr}\right)}^{\tau } $$

where *E* is the local constant modulus of elasticity of the rigid component of the system (Sahimi 1995). To a microscopic level, scaling functions of the modulus of elasticity can be used to determine the effective modulus of elasticity to macroscopic level. These scaling functions, determined mathematically as power law, can be written as

3a $$ E\ast =265.83{e}^{11.66\left(p-{p}_{cr}\right)}{\left(\frac{\left(p-{p}_{cr}\right)}{2{e}^2}\right)}^{\tau };\ \mathrm{M}\mathrm{P}\mathrm{a},\ \mathrm{f}\mathrm{o}\mathrm{r}\ \mathrm{a}\ \mathrm{squared}\ \mathrm{network} $$

and the effective modulus of elasticity as

3b $$ E\;{\ast}_{ef1}=E\ast +0.35 $$

4a $$ E\ast =21.64{e}^{16.75\left(p-{p}_{cr}\right)}{\left(\frac{\left(p-{p}_{cr}\right)}{e^{1.5}}\right)}^{\tau };\ \mathrm{M}\mathrm{P}\mathrm{a},\ \mathrm{f}\mathrm{o}\mathrm{r}\ \mathrm{a}\mathrm{n}\ \mathrm{hexagonal}\ \mathrm{n}\mathrm{etwork} $$

and the effective modulus of elasticity as

4b $$ E\;{\ast}_{ef2}=E\ast +0.32 $$

**Figure 3:**
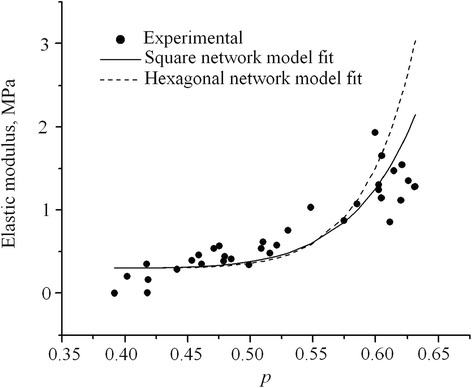
**Comparative results of the modulus of elasticity obtained via the hybrid model and lab experimental results as a function of** ***p*** **.**

Comparative plots for the mean value of the modulus of elasticity obtained with both nets and experimental results obtained from the soils mechanics lab are shown in Figure 3. Experimental data were determined for the possible porosity ranges in compacted soils. This compactness exists in Nature and can be reproduced in the laboratory (Born and Huang 1954). However computational models for both networks were run further the lab limits of maxima and minima porosities. This was done in order to identify easily the values of *p*_*cr*_*.*

The value of *p*_*cr*_ experimentally determined was 0.3943, lower than the geometric percolation threshold for a 2D squared net (*p*_*cr*_ = 0.5) and much less than the 2D hexagonal net (*p*_*cr*_ = 0.6527). Soil porosity is represented by (1-*p*) where *p* is ratio of the occupied network by soil solids. Soil critical porosity for a minimum stiffness *E* is given by 1-*p*_*cr*_. Displacements at the experimental percolation threshold related to geometric percolation of networks are due basically to dimensionality. Experimental percolation threshold was determined in soil samplings located in a 3D space, whereas model nets were considered in 2D. A non percolating aggregate in 2D could be the cross section of a percolating aggregate in 3D. This could explain why percolation threshold for 3D nets are much less than those similar in 2D, because a lesser amount of connected bonds are needed to form a percolating aggregate. Although working with 2D nets could seem very simplified, limitations in memory and processing speed of available hardware are an important factor to select the type of net for approximating soil structure. This is due to the number of degrees-of-freedom needed for the stress–strain analysis. In 3D, even reducing the number of degrees-of-freedom, networks shall be smaller. Even with these limitations, and taking into account the displacements of the percolation threshold in the predicting equations (3a, 4a), acceptable results are obtained. The degree of approximation based upon a statistical analysis of the results of the proposed model is 78%. This is considered a reliable level if one considers that traditional models of soils mechanical behavior have a level less than 50%.

## 5. Conclusions

Although percolation models appeared in the 1950’s, their use in soil mechanics has been limited mainly to the analysis of hydraulic conductivity. In porous media, transport phenomenon and electric conductivity have also been studied. Nevertheless, their application to the mechanical properties of soils has been scarce. In this work, percolation theory was resorted to the study of the soil stress–strain behavior. A model of regular nets was proposed to predict the effective modulus of elasticity via micro-structural properties such as layout, size, and shape of clay particles. Specifically, and as a result of this study, it can be concluded that squared and hexagonal nets can be used to model the soil stress–strain behavior of soils, compacted with natural humidity and different degrees of compactness. Nets were subjected to external loadings and it was assumed that each loop had a single elastic behavior. It was also considered that the loading transfer occurs through the contact points of grains; this approach neglects stresses of pores. Thus, a two-phase composite arises, namely, a solid phase due to clay grains and the porous phase with air inside the pores. The hybrid model (percolation-FEM) has been successfully used herein. From this study, and comparing results to reported results, the squared net seems to fit much better laboratory data for remolded soils. The prediction model was calibrated from soil elastic properties determined in the laboratory. The accuracy of the model for predicting the effective modulus of elasticity is about 72% for a squared net and 70% for a hexagonal one.

The percolation threshold for experimental data was less than any of the two models with regular nets. This means that stresses in natural soil are not transmitted only by the contact between grains, represented as nodes in the nets. It is conjectured that inter-particles sliding, associated to the viscous effect, traditionally neglected, is responsible for the difference. The shift of the percolation threshold is necessarily associated to the viscous effect which, in turn, is due to the different relaxation times for the two soil phases, the solid phase and the liquid phase. In the models, the liquid phase was not considered, thus the percolation threshold coincides with the percolation threshold for squared and hexagonal nets. In laboratory tests, since the liquid phase was not isolated, it was included in the percolation process. The soil takes most of the load, whereas the liquid phase takes longer in dissipating the load.

It is important to underline that the shift of the natural percolation threshold is strongly associated to dimensionality. Indeed this was determined via soil test probes in 3D and the percolation thresholds are smaller in this dimension than in the plane. The simplification of a 2D analysis is justified by the limitations of processing equipment and the information availability.

Even though soil porosity is much greater that the critical porosity in geometric nets, it keeps standing the loads without collapsing. Although the critical elasticity exponent τ is universal, which means it is independent from the net geometry and depends only on dimension, the scaling functions are not universal?? Or are not independent??. However, they may be set for predicting the value of the final effective modulus of elasticity, which is very important for the analysis and design of foundations on saturated and unsaturated soils. It is interesting to highlight that the soil natural porosity is very close to the percolation threshold. Physically, the critical porosity is the minimum necessary for reaching the soil strength.
